# Barriers and Facilitators Impacting Physical Activity Among Rural American Indian Older Adults

**DOI:** 10.1093/geroni/igab046.2881

**Published:** 2021-12-17

**Authors:** Maja Pedersen, Kari Harris, Jordan Lewis, Mattea Grant, Chelsea Kleinmeyer, Ashley Glass, Blakely Brown, Diane King

**Affiliations:** 1 Stanford University, Stanford, California, United States; 2 University of Montana, Missoula, Montana, United States; 3 Memory Keepers Medical Discovery Team/University of Minnesota Medical School, Duluth campus, Duluth, Minnesota, United States; 4 Confederated Salish and Kootenai Tribal Health Department, St Ignatius, Montana, United States; 5 University of Alaska Anchorage, Anchorage, Alaska, United States

## Abstract

Background. American Indian (AI) older adults experience pronounced health disparities and demonstrate among the lowest levels of physical activity (PA) of racial and ethnic groups. Nearly half of AI older adults live in rural areas, indicating distinct challenges to participation in PA. Research to identify factors influencing PA among this population is missing from the literature, yet is critical to inform culturally relevant PA intervention development and implementation. Purpose. To identify barriers and facilitators to PA among rural AI older adults using the ecological model and qualitative methods. Methods. A community-based approach was used to conduct semi-structured interviews with rural AI older adults. Interview questions were based on a multi-level ecological model. Content analysis was performed, using an iterative coding process to identify findings. Results. Participants’ (n=21) mean age was 66 years. Barriers and facilitators to PA were identified across ecological model levels. Barriers included factors such as caregiving and community responsibilities, lack of acceptable areas for walking, and overall lack of community-level support for older adult health. Facilitators included a personal connection to the land and ancestors through PA, multigenerational participation, and supportive tribal policies. Conclusion. This study addressed a critical gap in the literature by identifying barriers and facilitators among rural AI older adults, which can inform PA intervention development. In this way, their voices are uplifted to shape efforts addressing longstanding health disparities through relevant public health interventions.

